# The Potential Effects of Phytoestrogens: The Role in Neuroprotection

**DOI:** 10.3390/molecules26102954

**Published:** 2021-05-16

**Authors:** Justyna Gorzkiewicz, Grzegorz Bartosz, Izabela Sadowska-Bartosz

**Affiliations:** 1 Laboratory of Analytical Biochemistry, Institute of Food Technology and Nutrition, College of Natural Sciences, Rzeszow University, 4 Zelwerowicza Street, 35-601 Rzeszow, Poland; justyna5914@o2.pl; 2 Department of Bioenergetics, Food Analysis and Microbiology, Institute of Food Technology and Nutrition, College of Natural Sciences, Rzeszow University, 4 Zelwerowicza Street, 35-601 Rzeszow, Poland; gbartosz@ur.edu.pl

**Keywords:** phytoestrogens, neuroprotective effect, isoflavones

## Abstract

Phytoestrogens are naturally occurring non-steroidal phenolic plant compounds. Their structure is similar to 17-β-estradiol, the main female sex hormone. This review offers a concise summary of the current literature on several potential health benefits of phytoestrogens, mainly their neuroprotective effect. Phytoestrogens lower the risk of menopausal symptoms and osteoporosis, as well as cardiovascular disease. They also reduce the risk of brain disease. The effects of phytoestrogens and their derivatives on cancer are mainly due to the inhibition of estrogen synthesis and metabolism, leading to antiangiogenic, antimetastatic, and epigenetic effects. The brain controls the secretion of estrogen (hypothalamus-pituitary-gonads axis). However, it has not been unequivocally established whether estrogen therapy has a neuroprotective effect on brain function. The neuroprotective effects of phytoestrogens seem to be related to both their antioxidant properties and interaction with the estrogen receptor. The possible effects of phytoestrogens on the thyroid cause some concern; nevertheless, generally, no serious side effects have been reported, and these compounds can be recommended as health-promoting food components or supplements.

## 1. Introduction

Phytoestrogens are polyphenolic and non-steroid compounds that naturally occur in more than 300 plants. These compounds have a biological activity similar to the main female sex hormone, 17-β-estradiol (estra-1,3,5(10)-triene-3,17α-diol, 17α-E2, 17-epiestradiol). Owing to the structural similarity, phytoestrogens can bind to the estrogen receptors (ERs) and exert anti-estrogenic or pro-estrogenic effects. Phytoestrogens are distinguished by their pro-health effects, including the reduction of intensity of some symptoms of menopause, such as hot flushes, and the risk of osteoporosis, cardiovascular disease, obesity, metabolic syndrome, and type 2 diabetes, as well as of breast, prostate, and intestine cancer [1,2,3,4,5,6,7,8]. Most phytoestrogens are antioxidants [9,10], and their antioxidant properties may contribute to their pro-health effects; however, the main mechanism of their action is due to ER binding [11,12]. Phytoestrogens are used worldwide as an alternative to the estrogen replacement therapy (ERT) and can be administered as dietary supplements.

Four groups of phenolic compounds are classified as phytoestrogens: stilbenes, coumestans, lignans, and isoflavones [10]. The main natural stilbene is resveratrol (the *trans* isomer shows estrogenic activity), found mainly in grapes and peanuts. Resveratrol is synthesized in the grape skin; thus, red wines, fermented with skins are particularly rich in resveratrol [13]. Among coumestans, only some compounds (e.g., coumestrol) have estrogenic activity. Coumestrol is present mainly in legumes, but also in other vegetables, such as spinach or Brussels sprouts [14]. Lignans are a large group of polyphenols found in plants, especially in flaxseed, but also in wheat, tea, and fruits. They are metabolized to enterolignans (mammalian lignans). A representative compound is the non-estrogenic matairesinol, which is transformed by gut microflora to being estrogenic and easily absorbed enterolactone [15].

**Figure 1:**
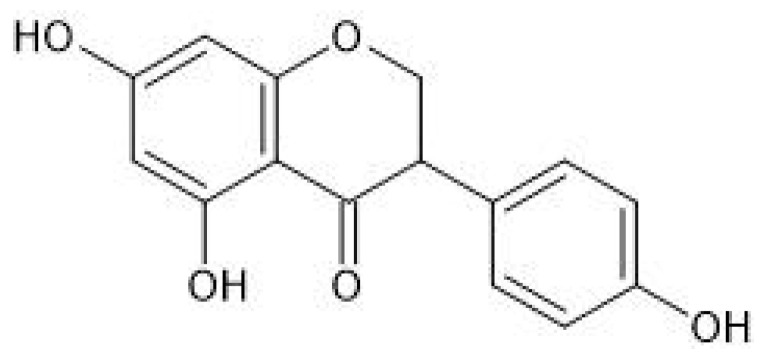
Chemical structure of isoflavones.

**Figure 2:**
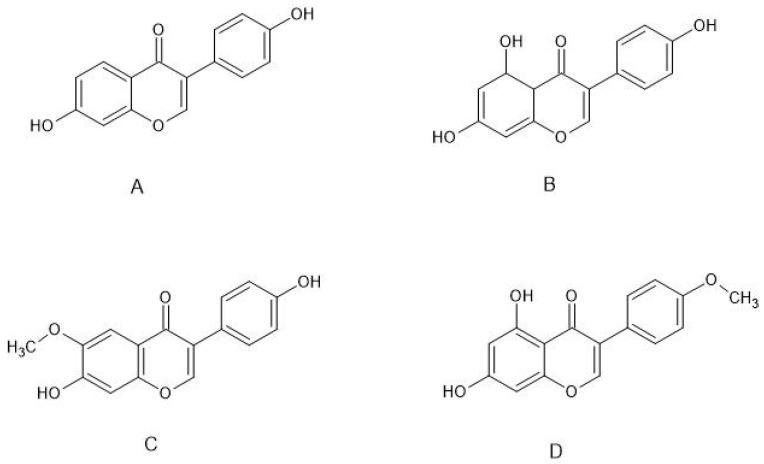
Structure of the main estrogenic isoflavones: (**A**) daidzein, (**B**) genistein, (**C**) glycitein, (**D**) biochanin A.

Isoflavones (Figure 1) are produced almost exclusively by plants of the *Fabaceae* family. Their main source is soybeans, but they are also present in other legumes, e.g., in red clover. The best known isoflavones are: daidzein, genistein, glycitein, and biochanin A (BCA) (Figure 2).

Isoflavones are the most extensively investigated phytoestrogens; therefore, in this short paper, we will refer mainly to isoflavones when using the term “phytoestrogens”.

Phytoestrogen intake is the highest in the East and Southeast Asia (about 20–50 mg/day) [16]. In Europe, where the consumption of soy products is much lower, typical values of phytoestrogen intake are 0.63–1.00 mg/day in men and 0.49–0.66 mg/day in women [17]. There are several reports on the feminizing effects of phytoestrogens (isoflavones) in men, such as lowered testosterone levels and increased estrogen levels. However, a more recent study could not confirm any significant effects of soy or isoflavone intake on the levels of reproductive hormones in men [18].

The structural similarity of phytoestrogens to 17-β-estradiol (E2) enables them to induce an antiestrogenic effect by binding to the ER. Two receptor subtypes have been detected in mammals; the estrogen receptor-α (ERα) (NR3A1) and the estrogen receptor-β (ERβ) (NR3A2) [19,20]. In humans, both receptor subtypes are ubiquitously expressed and control important physiological functions in various systems, including the cardiovascular, skeletal, reproductive, and central nervous systems. ERα is present mainly in the mammary glands, uterus, and thecal cells of the ovaries in females; in the testes, epididymis, and prostate stroma in males; and in the liver, bones, and adipose tissue. ERβ is found mainly in the prostate epithelium, bladder, adipose tissue, granulosa cells of the ovaries, the colon, and immune system. Both subtypes are prominently expressed in the cardiovascular and central nervous systems [21,22]. ERβ seems to play a minor role in mediating estrogen action in the uterus, on the hypothalamus/pituitary, and the skeleton, but seems to be important in the ovary, cardiovascular system, and brain [21,23].

Both receptor subtypes were reported to significantly affect gene expression in cancer cells [24,25]. ERα was found to either stimulate or inhibit the progression of cancer. A stimulant effect of ERβ on cell proliferation mediated by ERα has been postulated [26]. However, it was also reported that ERα and ERβ exert opposite effects on apoptosis, migration, and proliferation, and differentially influence the progression of cancer [25].

The mode of action of a phytoestrogen as an agonist/antagonist may depend on the endogenous estrogen content [27]. In recent years, the estrogenic activity of several phytoestrogens, in terms of receptor binding, has been quantified in vitro [28,29,30,31,32].

This review summarizes the current knowledge, mainly regarding the neuroprotective effects of phytoestrogens.

## 2. Neuroprotective Effects of Selected Phytoestrogens

In neurological studies conducted with the use of phytoestrogens, mainly soy isoflavones, it has been substantiated that estrogens may positively affect the proper functioning of the brain. The brain controls the secretion of estrogen (the hypothalamus-pituitary-gonads axis) and has an effect on estrogen-dependent processes in the body. The activation of the two nuclear ERs with selective agonists affects the levels of monoamines and their metabolites in brain areas, and play main roles in cognitive as well as affective functions. 17β-estradiol and the ERα agonist increased norepinephrine in the cortex, while ER ligands increased it in the ventral hippocampus. Changes in levels of the noradrenergic metabolite, 3-methoxy-4-hydroxyphenylglycol, and the dopaminergic metabolite, 3,4-dihydroxyphenylacetic acid, were noted in brain areas of ER ligand-treated animals (ovariectomized rats). 17β-estradiol increased the levels of 5-hydroxyindoleacetic acid in the brain. Moreover, 17β-estradiol and ERβ agonists increased the levels of the dopaminergic metabolite, homovanillic acid, following fenfluramine treatment [33].

It has not been unequivocally established whether estrogen therapy has a protective effect on brain function [34]. Despite the positive results found in some studies, around half of reports suggest zero effects [35]. Zhao et al. reported some neuroprotective effects of phytoestrogens; however, these effects might be due to the antioxidant action of phytoestrogens rather than binding to ER. Similar effects have been observed for other antioxidants, but it was considered doubtful whether phytoestrogens reduce the risk of Alzheimer’s disease (AD) or improve memory function in postmenopausal women [36].

The studies carried out so far have shown that consumption of soy isoflavones has a positive outcome on neurons in vivo in rodents models [37,38,39,40], whilst high-dose consumption may have a negative effect on the brain.

Genistein has anti-inflammatory, antioxidant, and anti-apoptotic properties; it can also exert a neuroprotective effect in AD. It was shown that the administration of high doses of genistein (20 mg/d) to rats increased the level of lactate dehydrogenase (LDH; the enzyme at the end of the metabolic chain of anaerobic glycolysis) in rat brain tissue, while a dose 2 mg/d of genistein decreased the level of LDH. DNA fragmentation was also detected in the brains of rats administered any amount of genistein. These results indicate that increased amounts of genistein contribute to the induction of cytotoxicity. It was shown that genistein also reduced the expression of the caspase-3 precursor and increased the level of cleaved caspase-3 in homogenates of rat brain tissues and in primary cultures in cortical neurons. Such results may indicate that prolonged administration of genistein in increased doses may contribute to cytotoxicity and apoptosis in brain tissues [41].

In vitro studies carried out by Gamba et al., and performed on human neuronal cell lines (SK-N-BE and NT-2), demonstrated that genistein prevents 24-hydroxycholesterol’s pro-oxidant effect and the potentiation of Aβ-induced necrosis and apoptosis. The action of this compound depends on whether there is a local increase in the level of reactive oxygen species (ROS), mainly hydrogen peroxide, which contributes to the redox imbalance of neurons [42].

Zhao et al. showed that genistein was neuroprotective in a SOD1-G93A transgenic mouse model of amyotrophic lateral sclerosis (ASL), suggesting that genistein could be a promising treatment for human ALS. These studies showed that genistein administration suppressed the production of pro-inflammatory cytokines and alleviated gliosis in the spinal cord of SOD1-G93A mice. The administration of genistein induced the autophagy process and contributed to the increase in the vitality of the spinal motor neurons. Genistein alleviated the symptoms of disease and prolonged the lifespan of SOD1-G93A mice [43].

Xu et al. found that genistein stimulates the production of brain-derived neurotrophic factor (BDNF) in an ER-dependent manner in cultured rat astrocytes (the most dominant and functional type of neuroglial cell) [44]. Pan et al. reported that genistein increased the viability of H19-7/IGF-IR neural cells via the upregulation of the synthesis of BDNF [45]. Genistein also protected SK-N-SH neuroblastoma cells against the toxicity of 6-hydroxydopamine; in this case, the underlying mechanism involved activation of the insulin-like growth factor-I receptor [46].

Cai et al. demonstrated the protective effect of genistein on Aβ_25–35_ -induced PC12 cell injury and on the CaM-CaMKIV signaling pathway. In vitro studies on PC12 cells also showed that Aβ_25–35_ decreased the cell survival rate compared to the control group. Genistein could significantly improve the PC12 cell survival rate, reduce the cell damage and apoptosis, and significantly down-regulate the expression of mRNA and the protein levels of CaM, CaMKK, CaMKIV, and tau protein in this cellular model of AD. Therefore, it was suggested that genistein had a neuroprotective effect in this AD model and that the mechanism of this effect may be related to the down-regulation of the CaM-CaMKIV signaling pathway and tau protein expression [47].

Phytoestrogens (genistein and BCA) protected cultured neuronal cells in a cellular model of brain ischemia (oxygen and glucose deprivation and resupply), through modulation of autophagy. A dual role of phytoestrogens in the regulation of autophagy has been proposed: stimulation of initiation of autophagy when autophagy has a pro-survival role, and inhibition of autophagy initiation when autophagy plays a pro-death role [48].

In ovariectomized rats challenged with pentylenetetrazole (inducting behavioral and neurochemical deficits) intraperitoneally, oral administration of genistein resulted in an improvement in the state of oxidative stress and ER expression. This effect can be attributed to the estrogenic, antioxidant, and/or anti-apoptotic properties of genistein [49].

Jiang et al. reported that genistein reduced apoptosis in the hippocampus, reduced the expression of proapoptotic factors (Bad, Bax, and cleaved caspase-3), and increased the expression of Bcl-2 and Bcl-xL. What is more, genistein effectively upregulated cAMP levels and the phosphorylation of cyclic AMP response element-binding protein (CREB) and TrkB, leading to activation of cAMP/CREB-BDNF-TrkB signaling. Genistein administration improved the general behavior and enhanced the learning and memory in the rats. These observations reveled that genistein exerts neuroprotective effects by suppressing isoflurane-induced neuronal apoptosis, as well as by activating cAMP/CREB-BDNF-TrkB-PI3/Akt signaling [50]. Other studies showed protective effects of genistein against SH-SY5Y cell damage induced by β-amyloid 25–35 peptide (Aβ_25–35_). Genistein increased the survival of SH-SY5Y cells, decreased the level of apoptosis, and reversed the changes in amino acid transmitters. The results suggested that genistein protects cells against Aβ-induced cytotoxicity, probably by regulating the expression of apoptosis-related proteins and Ca^2+^ influx through ionotropic glutamate receptors [51]. According to more recent evidence, the protective effect of genistein is associated with the inhibition of Aβ-induced Akt inactivation and Tau hyperphosphorylation [52].

Wei et al. described the effect of daidzein in a intracerebroventricular–streptozotocin (ICV–STZ)-induced rat AD model. Daidzein treatment led to improvement in ICV–STZ-induced memory and learning impairments. Furthermore, it restored the alterations in malondialdehyde, catalase, superoxide dismutase, and reduced glutathione levels [53].

Subedi et al. showed that a metabolite of daidzein, namely equol, protects neurons from neuroinflammatory injury, mediated by LPS-activated microglia. This metabolite of daidzein, which is formed by the human intestinal microflora, protects against neuroinflammatory damage by downregulating neuronal apoptosis. These results suggest that equol is a potential neuroprotective nutraceutical, by regulating the state of neuritis [54].

Phytoestrogens exert direct effects on androgen receptors in the brain and together with ER actions may modulate the neural circuit functions. Male mice treated with a low phytoestrogen diet demonstrated a reduction in activation of second messengers correlated with plasticity in the hippocampus synapse. This diet induced a profound decrease in long-term potentiation (LTP) in the ventral hippocampus, altered territorial marking behavior, a reduction of intermale aggression, and a general disturbance of social behavior. Additionally, acute perfusion of equol was able to rescue this LTP deficit, demonstrating a possible modulation by phytoestrogen of the hippocampus plasticity, as well as memory function [55].

Central nervous system diseases are quite common. Alzheimer′s disease is a disease that leads to memory loss and even cognitive decline. The disease causes a build-up of a protein substance called Aβ in the brain. Amyloid prevents the function of the neurons concerned, thus hindering communication, among other things. Resveratrol reduces the action of Aβ proteins by stimulating their breakdown through a proteasome mechanism. It has been proven that a diet rich in resveratrol in mice with symptoms of AD slows down the progression of AD [56,57].

The changes in Parkinson′s disease (PD) are caused by the death of gray matter cells in the brain and the atrophy of the cerebral cortex. The reduction or inhibition of dopamine production, resulting in an imbalance in the cholinergic–dopaminergic neurons in the brain, contributes to this effect. Karlsson et al. [58] showed that resveratrol protects mesenchymal embryonic cells from mice against *tert*-butyl-hydrogen peroxide by removing the free radicals formed. Hunter et al. reported that inflammation supports the development of PD. It has been shown that resveratrol has a protective effect on cells because it inhibits COX-2 cyclooxygenase, an enzyme that catalyzes the synthesis of compounds involved in the inflammatory process. This compound also reduces the activity of tumor necrosis factor [59].

In turn, in the studies of Sarfraz et al., BCA also showed anti-inflammatory, anticancer, neuroprotective, antioxidant, and anti-microbial properties, which help to combat cancer development via apoptosis induction, inhibition of metastasis, and cell cycle arrest. Biochanin A fights inflammation by blocking the expression and activity of pro-inflammatory cytokines via modulation of NF-κB and mitogen-activated protein kinases (MAPKs). What is more, BCA is neuroprotective, contributing to the inhibition of apoptosis of neurons [60].

El-Sherbeeny et al. showed that BCA protected dopaminergic neurons against rotenone-induce damage by ameliorating the oxidative burden and neuroinflammation. BCA treatment improved motor function of rotenone treated mice in the pole tests. The mechanism that uses BCA causes, among other things, lessened levels of proinflammatory cytokines and increased phosphorylation of phosphoinositide 3-kinase/Akt protein kinase/mechanistic target of rapamycin (PI3K/Akt/mTOR) signaling pathway proteins. The phytoestrogen activates PI3K/Akt/mTOR signaling, leading to the protection of dopaminergic neurons [61].

Guo et al. found that BCA protected rats against brain ischemic injury due to its antioxidant action and inhibition of inflammation. The activation of the Nrf2 pathway and the inhibition of the NF-κB pathway may contribute to the neuroprotective effects of BCA. Pretreatment with BCA significantly decreased the size of brain infarct size and the extent of edema. Biochanin A also enhanced the activities of the main antioxidant enzymes, superoxide dismutase and glutathione peroxidase [62]. Khanna et al. showed that biochanin A was a potent inducer of glutamate oxaloacetate transaminase (GOT) gene expression in neural cells. Phytoestrogen significantly increased GOT mRNA and protein expression and protected against glutamate-induced cell death. BCA mitigated stroke-induced injury by inducing GOT expression. The phytoestrogen had a neuroprotective effect and prevented the formation of a stroke state [63].

Schreihofer and Redmond demonstrated that pretreatment with dietary levels of soy phytoestrogens (genistein, daidzein, and the daidzein metabolite equol) may mimic the neuroprotective effects observed with estrogen and appears to use the same ER-kinase pathways to inhibit apoptotic cell death [64].

Various lignans found in plant cell walls and fiber-rich foods and seeds, had a positive effect on cognition and markers of AD induced in mice [65,66,67,68,69]. It has been shown that higher dietary lignan intake may be associated with better cognitive function [70,71], while no improvement in cognitive performance was observed when consuming coumestrol [72]. However, dietary intake of isoflavones showed no association with cognition [70].

Recent studies have demonstrated a novel mode of phytoestrogen action, via regulation of autophagy. Autophagy is a fundamental cellular mechanism enabling the removal of nonfunctional proteins and organelles. Phytoestrogens may either promote or inhibit the initiation of autophagy, depending on whether stimulation of autophagy results in cell survival or cell death. These data suggest the therapeutic potential of phytoestrogens in brain ischemia based on the modulation of autophagy [48].

In conclusion, the data regarding the beneficial effects of phytoestrogens on neurological health appear to be inconclusive.

## 3. Other selected Applications of Phytoestrogens

### 3.1. Phytoestrogens in Postmenopausal Indications

There have been many studies that observed that menopausal vasomotor symptoms, such as hot flushes and sweating, are common symptoms during menopause and contribute to physical discomfort [73]. When estrogen levels decline during the menopause, it influences the development of obesity, the plasma lipid profile, and platelets [74,75]. Miller and others evaluated the relationship between overweightness or obesity and the metabolism of daidzein isoflavone to equol or *O*-desmethylangolensin (ODMA). More than half of the women did not produce ODMA, which is associated with obesity in peri- and post-menopausal women [76].

Ribeiro et al. conducted a randomized controlled trial in postmenopausal women, who were administered an oral extract of glycine alone, or isoflavone with a probiotic or hormone therapy (with the use of estradiol and norethisterone acetate). The vaginal health score increased in the isoflavone and hormone therapy groups. Probiotics improved the metabolism of the isoflavones treatment. However, the increase in the contents of isoflavones failed to exert an estrogenic effect on the urogenital tract [77].

Felix et al. compared the therapeutic properties of BCA against 17-β estradiol replacement therapy in zymosan-induced arthritis (ZIA) in mice. They noticed that BCA’s anti-inflammatory effect is higher than that of ERT. Zymosan induced paw edema in mice was inhibited by pre-treatment with BCA, which attenuated neutrophil accumulation. What is more, this isoflavone had an anti-inflammatory effect, similar to 17-β estradiol, especially in ZIA. These results indicate that BCA could be potentially useful in the treatment of postmenopausal arthritis [78].

Mohamed et al. demonstrated the effect of anastrozole (ANA), BCA in monotherapy, and BCA + ANA on the degree of development of bone loss in ovariectomized rats. Biochanin A was shown to alleviate the effects induced by ANA, which can worsen osteoporosis in bilaterally ovariectomized female rats. These findings suggest that BCA may be a promising supplement for bone health [79].

### 3.2. Phytoestrogens and Cardiovascular Health

Several studies have shown that estrogen deficiency often contributes to the development of cardiovascular diseases in women, and it has been proven that phytoestrogens can contribute to reducing this risk. Phytoestrogens can both protect and counteract the formation of atherosclerotic plaque, crucial for arterial pathogenesis in many cardiovascular diseases. The health-promoting effect of isoflavones on the cardiovascular system has been demonstrated at an experimental and clinical level [80]. Clinical studies by Schouw et al. and Kokubo et al. have shown a positive relationship between isoflavone consumption and the elimination of cardiovascular diseases compared to humans tested before isoflavone administration [81,82]. Studies have shown that consumption of isoflavones reduced the risk of cerebral infarction and myocardial infarction in women, especially in postmenopausal women [82].

### 3.3. Phytoestrogens in Cancer Prevention

Many researchers have attempted to study the effects of phytoestrogens on breast cancer cells in women. The compounds used in the experiments were soybean ingredients; the studies were carried out both in men with prostate cancer and in women with breast cancer [83]. In the clinical trials that have been carried out, it was observed that through their estrogenic and proliferative effects, phytoestrogens may increase the incidence of breast cancer in more sensitive individuals [84,85]. In studies conducted on women who followed a diet rich in soy, a reduction in the risk of breast cancer was observed [86,87,88,89]. Fritz et al. [90] reviewed the potential effects of consumption of soybeans, red clover, and isoflavones on the incidence and recurrence of breast cancer. About 40 randomized controlled trials and 80 observational studies were analyzed. This analysis led to the conclusion that soy consumption may lower the risk of breast cancer, recurrence, and mortality. The involvement of equol has also been detected and it was postulated that this compound may have a beneficial effect in reducing the incidence of breast cancer [91,92]. However, several studies presented controversial results, showing the absence or presence of the favorable equol effects. It is known that between 30 and 40% of the population has the ability to convert daidzein to equol. When also taking into account in vitro studies, it can be concluded that equol is more biologically active than its parent compound daidzein and that the variability of daidzein effects may be related to the variable gut microflora, resulting in inter-individual differences in the daidzein conversion to equol [93].

The influence of lignans, enterodiol, and enterolactone on the occurrence of breast cancer was also investigated, suggesting their protective potential due to mechanisms both dependent and independent of estrogen receptors [94,95,96,97,98,99].

Epidemiological studies in Japan and clinical trials have found that isoflavone consumption may be associated with a reduced risk of lung cancer [100]. It seems that consuming soy foods lowers the risk of lung cancer [101]. Subsequent follow-up studies revealed the effect of a higher serum isoflavone concentration in reducing the risk of stomach cancer [102]. Other studies have shown a positive effect of reducing the risk of prostate cancer by consuming food rich in soy, genistein, and daidzein [103,104,105]. Epidemiological studies have shown that phytoestrogen diets in pre- and post-menopausal women reduce the risk of thyroid cancer [106,107]. Moreover, women’s diets rich in isoflavones or soy reduce the risk of endometrial and ovarian cancer [108,109]. The plasma levels of isoflavones, especially genistein, have been found to be inversely correlated with several types of cancer, including prostate, lung, colorectal, and breast [100,110,111].

### 3.4. Thyroidal Effects of Phytoestrogens

Studies on soy isoflavones, daidzein, and genistein have shown their inhibitory effects in vitro on thyroid peroxidase (TPO), an enzyme involved in the synthesis of T3 and T4 [112]. In rats daidzein and genistein inhibited TPO activity in in vivo studies [113]. It has been suggested that estrogens have an indirect effect on the thyroid function, which raises concerns that phytoestrogens may adversely affect thyroid function. However, clinical studies on the effects of soy isoflavones on thyroid function, reviewed by the European Food Safety Authority [114], have not been conclusive. In some cases, risk factors including iodine deficiency may increase people’s susceptibility to the potentially adverse effects of soy isoflavone on thyroid function [115,116].

## 4. Conclusions

The use of phytoestrogens in the diet has benefits; however, it has also some limitations. Taking foods rich in phytoestrogens reduces the risk of symptoms in menopause, cardiovascular disease, and many types of cancer, including prostate cancer and uterine cancer. Reports on their neuroprotective effects concern the protection of neural cells against injury evoked by various factors and the beneficial effects in animal models of AD and PD. Clinical trials have generally indicated no serious side effects. However, in many cases the results are controversial, and the neuroprotective and other beneficial effects of phytoestrogens require further studies.
